# The role of vitamin D in autoimmune diseases: could sex make the difference?

**DOI:** 10.1186/s13293-021-00358-3

**Published:** 2021-01-12

**Authors:** Maria Luisa Dupuis, Maria Teresa Pagano, Marina Pierdominici, Elena Ortona

**Affiliations:** Center for Gender Specific Medicine, Istituto Superiore di Sanità, Rome, Italy

**Keywords:** Vitamin D, Autoimmunity, Multiple sclerosis, Rheumatoid arthritis, Systemic lupus erythematosus, Sex hormones, Estrogen, Gender, Sex

## Abstract

Over the last decades, a central role for vitamin D in immune modulation has been well established. The active form of vitamin D, i.e., 1,25-dihydroxyvitamin D, through the interaction with vitamin D receptor, exerts different activities on the innate and adaptive immune system, among which suppression of inflammation and promotion of tolerogenic responses. Vitamin D insufficiency has been linked to autoimmune disorders that commonly display significant differences between females and males due to genetic, epigenetic, hormonal, and environmental factors. Notably, a number of studies recently showed a cross-talk between vitamin D and the sex hormone estrogen. Estrogen-mediated effects on immune response may favor a Th1 profile or a Th2 profile, depending on hormone concentration. Thus, estrogen-mediated effects appear to be variable on autoimmunity depending on its concentration but also on the pathogenic mechanisms underlying the different autoimmune diseases (i.e., Th1- or Th2-mediated diseases). Notably, estrogen has been demonstrated to enhance vitamin D function favoring its accumulation, and increasing the expression of vitamin D receptor, thus resulting in a more potent anti-inflammatory response in females than males. On the other hand, vitamin D has been shown to downregulate in immune cells the expression of aromatase, which converts testosterone to estrogen, leading to a decrease in estrogen level. Overall, available data allow us to hypothesize a higher protective effect of vitamin D-based therapeutic approaches in women, at least in fertile age, than in men. Future studies are needed to expand current knowledge on the immunomodulatory role of vitamin D in a sex and gender perspective, paving the way to a more personalized therapeutic approach in autoimmune diseases.

## Introduction

Vitamin D is well known for its essential role in regulating calcium homeostasis and maintaining healthy bones [1]. Numerous studies suggest that vitamin D has many other effects, such as the modulation and regulation of innate and adaptive immune responses [2–4]. The majority of vitamin D is produced by the human body through the action of sunlight, while smaller amounts are obtained from dietary sources. Gender seems to affect 25-hydroxyvitamin D (25(OH)D) level, that represents the biomarker of vitamin D status, although contrasting results have been reported, some data showing higher 25(OH)D levels in males [5] and others in females [6]. However, some evidence suggests sex-specific determinants in the modulation of 25(OH)D level [7]. For instance, the time spent outdoors seems to have an effect on 25(OH)D mainly in women, whereas physical activity and smoking predict 25(OH)D level in men [7].

Vitamin D deficiency adversely affects bone mass causing rickets in children and adolescents, and osteoporosis and osteomalacia in adults. In addition, vitamin D deficiency has been also linked to the onset/maintenance of other diseases including cardiovascular diseases [8], chronic obstructive pulmonary diseases [9], allergic asthma [10], type 2 diabetes [11], and autoimmune diseases [12–14].

The two major types of vitamin D are vitamin D_2_ (ergocalciferol) and vitamin D_3_ (cholecalciferol). The vitamin D_2_ is mainly human-made and added to foods, whereas vitamin D_3_ is synthesized in the skin of humans from 7-dehydrocholesterol although it can also found in animal-based foods (like cod liver oil and oily fish); both forms function as prohormones.

The activation of vitamin D involves two enzymatic hydroxylation reactions. The first is mediated by the 25-hydroxylase (CYP2R1) that converts, in the liver, the vitamin D to 25-hydroxyvitamin D form (25(OH)D) [15]. The second reaction converts 25(OH)D to 1,25-dihydroxyvitamin D (1,25(OH)2D) or calcitriol, the active form, by 1α-hydroxylase (CYP27B1) in kidney or other organs [15]. CYP27B1 is induced by hypocalcemia and hypophosphatemia whereas hypercalcemia and hyperphosphatemia reduce its expression [16]. 1,25(OH)2D binds to vitamin D binding protein to be transported in the blood stream. 1,25(OH)2D is also the active ligand for the vitamin D receptor (VDR) which is present in many tissues and even in different cell types not involved in the regulation of calcium and phosphate metabolism, including immune cells such as dendritic cells (DCs), macrophages/monocytes, neutrophils, T and B lymphocytes. VDR is a transcription factor and member of the steroid hormone nuclear receptor family. After binding with 1,25(OH)2D, VDR heterodimerizes with the retinoid X receptor, forming a complex with high affinity for the vitamin D response element that modulates gene expression in a ligand-dependent manner [17].

Over the last decades, it has been demonstrated that VDR regulates the transcription of numerous genes involved in innate and adaptive immunity, exerting modulatory effects on the immune system [2]. The VDR gene may also present diverse polymorphisms that affect its function and some of these polymorphisms (for example FokI, BsmI, and TaqI) have been shown to predispose to autoimmune diseases, disorders characterized by an exaggerated immune response that leads to organ and tissue damage [18, 19]. Notably, autoimmune disease occurrence has been associated with low circulating levels of 25(OH)D [12]. Most autoimmune diseases are more prevalent in women than in men. Symptom severity, disease course, response to therapy, and overall survival may also differ between males and females with autoimmune diseases [20]. Genetic, epigenetic, sex hormones, and environmental factors are crucial determinants in affecting these differences [21, 22]. Regarding sex hormones (e.g., estrogens and androgens), their effects on autoimmunity appear to be diverse depending on the hormone level, the specific receptor types expressed in immune cells, and the pathogenic mechanism underlying the different autoimmune diseases [23]. For instance, androgens, in particular testosterone, display an anti-inflammatory and immunosuppressive role [24, 25], whereas estrogens display a dual effect depending on their concentration. In fact, high levels of estrogens (e.g., periovulatory or pregnancy levels) promote a shift from a pro-inflammatory T helper 1 (Th1)/Th17 immune response toward an anti-inflammatory Th2/T regulatory (Treg) response whereas low levels of estrogens (e.g., luteal or postmenopausal levels) induce an opposite shift [26, 27]. Thus, menarche, menses, pregnancy, postpartum period, menopause, and the use of hormone replacement therapies all impact disease activity [28]. For instance, pregnancy is associated with an increase in disease flares in the autoimmune disease systemic lupus erythematosus (SLE) due to the increased Th2 response and the enhanced production of pathogenic autoantibodies [29]. Conversely, high levels of estrogens during pregnancy have a protective effect in Th1-dominant autoimmune diseases, like multiple sclerosis (MS) and rheumatoid arthritis (RA) [21, 29].

Notably, growing evidence suggests a cross-talk between sex hormones and vitamin D. While studies on the interaction between androgens and vitamin D are at the beginning [30], the interaction between estrogens and vitamin D has been more widely investigated supporting the synergistic role of these hormones in sex bias of autoimmune diseases.

In this review, we summarize the impact of vitamin D in immunity and autoimmunity, with a special focus on its interaction with estrogens, taking into account the differential estrogen-mediated effects in autoimmune diseases. Three classical female-predominant autoimmune diseases, i.e., MS, RA, and SLE, characterized by different impact of estrogen-mediated effects on disease pathogenesis, will be analyzed in details.

### Vitamin D, sex hormones, and immune system

#### Vitamin D and immune system

The active form of vitamin D, 1,25(OH)2D, exerts immunologic activities on multiple components of the innate and adaptive immune system [2–4]. 1,25(OH)2D regulates innate immune cell subsets differentiation and maturation, antigen presentation, and production of cytokines and chemokines. In monocytes/macrophage, besides its direct antibacterial effect on transcription of antimicrobial peptides such as cathelicidin and β-defensin 2 [31], 1,25(OH)2D inhibits inflammation by suppressing the expression of Toll-like receptor (TLR)2/4 and the production of inflammatory cytokines such as interleukin (IL)-1, IL-6, and tumor necrosis factor alpha (TNF-α) that play a crucial pathogenic role in autoimmune diseases [32]. Several studies demonstrated that 1,25(OH)2D negatively regulates the differentiation, maturation and immunostimulatory ability of DCs by reducing the expression of major histocompatibility complex class II (MHC-II), CD40, CD80 and CD86 molecules and the maturation proteins CD1a and CD83 [33, 34]. Notably, in vitamin D receptor knockout (VDR KO) mice, absence of VDR results in increased maturity of lymph node DCs [33]. In addition, 1,25(OH)2D promotes a tolerogenic state decreasing the synthesis of IL-12 and type 1 interferon (IFN) and enhancing that of IL-10 [35]. Together, these effects of 1,25(OH)2D inhibit DC-mediated T cell activation. Regarding T lymphocytes, 1,25(OH)2D inhibits T cell proliferation and IFN-γ and IL-17 production whereas it enhances IL-4 and IL-10 production, favoring a switch from a Th1 and Th17 to a Th2 and Treg cytokine profiles [36]. Additionally, 1,25(OH)2D induces Treg differentiation [37].

Finally, 1,25(OH)2D influences the activation of B cells, the differentiation in plasma cells, and the production of antibodies. In particular, it suppresses the differentiation from mature B cells to plasma cells and class-switched memory B cells, also reducing immunoglobulin production including autoantibodies [38, 39]. As observed for DCs, vitamin D is able to downregulate the expression of the co-stimulatory molecule CD86 in B cells interfering with T cell activation [40]. This control on B cell function is clinically relevant in autoimmune diseases as B-cells producing autoantibodies play a crucial role in the pathophysiology of autoimmunity. The role for low serum 25(OH)D as a risk factor in autoimmunity is reinforced by the observation that healthy subjects, positive for antinuclear antibody (ANA), are significantly more likely to show low vitamin D serum levels than healthy subjects negative for these autoantibodies [41].

#### Estrogen and immune system

Estrogens, in particular 17-β estradiol (E2), can influence the immune system due to the presence of estrogen receptors on immune cells [42–44]. As stated above, estrogen effects depend on hormone concentration but also on the type of target cells and the estrogen receptor subtype expressed on a given cell type [23]. In particular, 17-β estradiol (E2), at high levels (e.g., periovulatory or pregnancy levels) exerts mainly anti-inflammatory actions such as (i) inhibition of the production of pro-inflammatory cytokines, e.g., TNF-α, IL-1β, and IL-6, (ii) induction of the expression of anti-inflammatory cytokines, e.g., IL-4, IL-10, and transforming growth factor (TGF)-β, (iii) promotion of Treg function, and (iv) inhibition of the migration of leukocytes, particularly neutrophils and monocytes, into inflamed areas [27, 45, 46]. At lower concentrations (e.g., luteal or postmenopausal levels), E2 stimulates TNF-α, IFN-γ, and IL-1β production and natural killer (NK) cell activity [27, 45, 46]. Both at high and low concentrations, E2 suppresses bone marrow B cell lineage precursors but enhances antibody production including autoantibodies [27, 45–48].

#### Cross-talk between vitamin D and estrogen

Some effects of vitamin D appear to be different in men and women and strictly related to its interplay with estrogens. Interestingly, studies in humans and in animal models showed that E2 is able to decrease the expression of CYP24A1, the cytochrome P450 component of the 25-hydroxyvitamin D(3)-24-hydroxylase enzyme, which inactivates vitamin D [49–51]. This effect leads to vitamin D accumulation, thus resulting in a more potent anti-inflammatory response in females than in males. Interestingly, the anti-inflammatory effects mediated by E2 in females could be reproduced treating immune cells from male subjects with this hormone [51]. In addition, E2 increases the expression of VDR gene in diverse human and rat tissues [52–54] and, in particular, in CD4^+^ T cells from mice [50]. The relationship between E2 and vitamin D is further supported by (i) the significant increase of 25(OH)D levels observed in women assuming estrogen containing contraceptives [55], and (ii) the association between low 25(OH)D levels and low E2 levels in women [56].

**Fig. 1:**
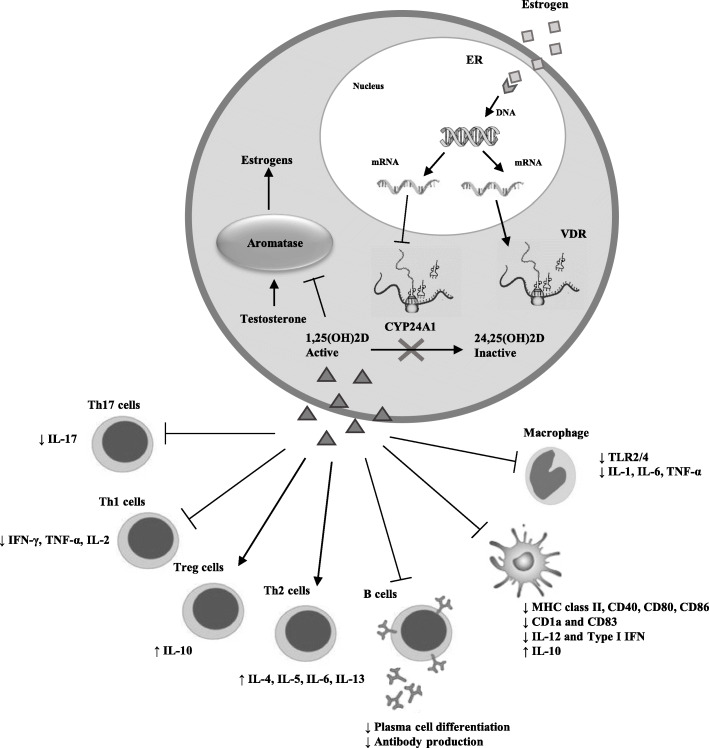
Cross-talk between vitamin D and estrogen and vitamin D effects on immune response. Estrogen decreases the expression of CYP24A1, which is involved in the inactivation of vitamin D, and increases the expression of VDR gene. 1,25(OH)2D downregulates aromatase expression, decreasing estrogen level. 1,25(OH)2D also regulates immune cell subsets, interfering with antigen presentation and cytokine production

On the other hand, 1,25(OH)2D affects in a tissue-specific way peripheral estrogen metabolism [57]. In mouse splenic T lymphocytes, 1,25(OH)2D enhances the expression of the CYP19 gene encoding aromatase, the enzyme that produces E2 from testosterone [50]. Vitamin D stimulation of CYP19 gene transcription has also been described in human glial cells [58], placental trophoblasts [59], and osteoblasts [60–62]. Conversely, 1,25(OH)2D has been observed to downregulate aromatase expression and inflammatory cytokines in human macrophages by a direct inhibition of its transcription via promoter II and, indirectly, inducing a decrease in the level and function of prostaglandins which are well-known stimulators of aromatase transcription [63, 64]. Figure 1 summarized the cross-talk between vitamin D and estrogen and its impact on immune response.

### Multiple sclerosis

Multiple sclerosis (MS) is an autoimmune neurodegenerative disease mediated by organ-specific T cells. Neurological dysfunction is due to the formation of focal lesions in the central nervous system (CNS) resulting in demyelination, loss of oligodendrocytes, and axonal damage [65]. Commonly, MS is characterized by exacerbations and remissions (relapsing-remitting) followed by a secondary progressive course. Less frequently, MS shows a primary progressive course. MS is three times more common in women than in men in most parts of the world, and the onset of the disease occurs earlier in females than in males. The sex ratio in MS appears to be increasing; this trend is observed mainly in relapsing-remitting MS and is linked to a latitudinal gradient suggesting that a complex interaction among genetic and epigenetic factors, sex hormones, and environmental factors takes place [66]. Even though women have a higher risk of developing MS as compared to men, female sex is associated with a better clinical outcome in relapse-remitting MS and a lower accumulation of disability compared to male sex [67]. During pregnancy a decrease of MS activity, followed by a rebound in the first 3 months postpartum, has been observed [68]. This effect is, at least partially, due to the estriol- and progesterone-induced immunological changes favoring a Th2 (anti-inflammatory cytokines) over a Th1 (pro-inflammatory cytokines) profile whereas the opposite happens in the postpartum period. In addition, disease severity seems to worsen after menopause [69]. Overall these epidemiological data suggest a protective role for E2 in MS, also confirmed by in vivo experiments in which this hormone inhibited clinical and histological signs of experimental autoimmune encephalomyelitis (EAE) in mice [70]. Growing evidence suggests that vitamin D is an environmental factor significantly affecting MS prevalence that is closely associated with the latitudinal gradient, where an increased exposure to UVB stimulates the cutaneous production of vitamin D reducing the risk of disease [71, 72]. Accordingly, low levels of 25(OH)D, in association with the presence of genetic polymorphisms involving the metabolism of vitamin D, imply a greater risk of developing MS [73–75]. In addition, it has been observed that the presence of gene variants of the CYP27B1 seems to increase the risk and activity of MS [76–78].

MS patients have lower serum levels of 25(OH)D than healthy subjects, associated with an increase in self-reactive T lymphocytes in the CNS. No differences have been demonstrated in vitamin D levels between female and male MS patients [51]. However, vitamin D deficiency seems to have a major effect in terms of increasing susceptibility to MS in women than in men [51]. In fact, in vitro treatment of CD4^+^ T lymphocytes from MS patients with 1,25(OH)2D reduced T cell proliferation as well as IFN-γ and IL-17 production, and increased IL-10 production, with a greater effect in lymphocytes isolated from female than male patients [51]. Notably, T lymphocytes from male MS patients, in the presence of 1,25(OH)2D plus E2, showed a significant decrease in proliferation rate and a significant increase in IL-10 production, similar to those observed in cells isolated from female patients. These data suggest a strict interaction between vitamin D and E2, further supported by studies in animal models of EAE. In this model, the administration of 1,25(OH)2D inhibited the induction of EAE in female mice but not in male mice or ovariectomized (OVX) female mice [79]. E2 was also able to restore vitamin D-mediated EAE protection in female OVX mice [79]. In addition, in EAE-induced female mice, vitamin D reduced demyelination, increased the production of IL-4, IL-10, and TGF-β, and reduced the production of IFN-γ, IL-6, TNF-α, and IL-17, thus favoring a shift from Th1 and Th17 to Th2 and Treg phenotypes [80]. Interestingly, in T lymphocytes from a mouse model of EAE, E2 was able to enhance the expression of *Vdr* gene and to suppress that of *Cyp24a1* gene which is involved in vitamin D degradation, thus prolonging its half-life [50]. Additionally, in this mouse model, E2 displayed a protective role against EAE in wild-type but not in mice lacking *Vdr* gene in CD4^+^ T cells, suggesting that E2 action is dependent on *Vdr*^+^CD4^+^ T cells [50]. E2 also increased Treg cells in a *Vdr*-dependent manner further supporting a synergy between E2 and vitamin D [50]. Overall, the cross-talk between vitamin D and estrogen suggests that therapeutic strategies based on vitamin D could be more effective in females than in males. In addition, deprivation of vitamin D and E2, present in menopausal women, could favor disease progression in this group of MS patients. Up to date, results from clinical studies regarding vitamin D supplementation, alone or in combination with other drugs, in MS patients are controversial [81]; some studies showing a beneficial effect of this hormone in decreasing relapse rate and ameliorating inflammatory markers and magnetic resonance imaging findings [82–85] whereas other studies showing no benefits on disease outcomes [86–92]. All the above-mentioned studies are underpowered and large randomized-controlled trials are required to better understand the effect of vitamin D supplementation in MS, stratifying data by sex, age, and stage of the disease. Studies in animal models and in humans on the effects of vitamin D in MS are summarized in Table 1.

**Table 1:** Studies in animal models and in humans on the effects of vitamin D in MS

Studies in animal models	Studies in humans
Inhibition of EAE-induction in female mice [79] ↓ demyelination [80] ↓ Th1, Th17 cells, ↑ Th2, Treg cells, ↑ IL-4, IL-10, TGF-β, ↓ IFN-γ, IL-6, TNF-α, IL-17 [80]	In vitro studies: ↓ T cells proliferation, ↓ IFN-γ, IL-17, ↑ IL-10 (higher effects in ♀ than in ♂) [51] Clinical studies: ↓ relapse rate and inflammatory markers [82, 84, 85] Improvement of MRI findings [83] No improvement in MS outcomes [86–92]

*EAE* Experimental autoimmune encephalomyelitis, *MRI* Magnetic resonance imaging, *MS* Multiple sclerosis. ↓ indicates decrease, ↑ indicates increase

### Rheumatoid arthritis

Rheumatoid arthritis (RA) is a chronic systemic inflammatory disease found in all geographical areas affecting 1–2% of the world population with an apparent reduction in the northern hemisphere [93, 94]. Symptomatology is characterized by the appearance of symmetric arthritis with the predominant involvement of small joints. Due to its systemic nature, the disease can affect other organs and systems with a wide variety of extra articular manifestations (e.g., rheumatoid nodules, vasculitis, and some cardiac dysfunctions) [94]. As observed in most autoimmune diseases, RA shows a higher incidence in females especially in the post-menopausal period (female:male ratio of 3:1). In addition, disease severity seems to be worse in women than in men [95, 96]. Women are more likely to display conditions like depression, fibromyalgia, osteoporosis, and thyroid dysfunctions than males. A key role in the pathogenesis of RA is played by Th1 and Th17 cells, which contribute to maintaining a chronic inflammatory state at the level of the joint synovium [97]. The etiology of RA is still unknown but, also in this instance, the interaction between genetic, epigenetic, hormonal, and environmental factors is believed to be fundamental in the development of the disease [94]. Regarding sex hormones, a multifaceted role in RA onset and severity has been revealed [46, 98]. The female to male ratio of 3:1 may suggest that estrogens increase the risk of RA. However, some data, such as (i) the peak incidence at age 45–55, which coincides with the perimenopausal years, (ii) the lack of association between hormonal therapy and the risk of developing RA, and (iii) the reduction of disease activity during pregnancy, support a systemic anti-inflammatory effect of estrogens. On the other hand, at local level, a pro-inflammatory role for estrogens in peripheral tissues of RA patients has been suggested [99–101]. In both male and female RA patients, estrogens are strongly upregulated in synovial fluid due to the increased aromatase activity in monocyte-derived macrophages, induced by local inflammatory cytokines (TNF-α, IL-6, IL-1). The upregulated estrogen concentrations detected in synovial fluid of RA patients are mainly characterized by the hydroxylated forms, in particular 16α-hydroxyestrone which displays a mitogenic activity, supporting the proliferative state of the synovial cells. Interestingly, as stated above, it has been demonstrated that vitamin D is able to decrease the expression of aromatase in human macrophages hampering local inflammation [63].

Thus, a vitamin D deficiency could contribute to synovial inflammation in RA, leading to downregulation of aromatase activity and increased synthesis of estrogens.

More generally, different studies in animal models and in humans support a role for vitamin D in RA development and severity. In animal models of RA, vitamin D was found to reduce the development and the severity of the disease, also being able to reduce anti-type II collagen antibodies (that are associated with inflammation, particularly at RA onset) and to shift the CD4+ differentiation from a Th1-Th17 to a Treg phenotype [102–105]. Consistent with in vivo studies, in vitro studies carried out in human immune cells suggest a protective role for vitamin D in RA. In monocyte-derived macrophages of RA patients, 1,25(OH)2D was able to reduce the production of pro-inflammatory cytokines (i.e., TNF-α, IL-1α, IL-1β, and IL-6) and receptor activator of nuclear factor κβ ligand (RANKL), a molecule that induces the differentiation and activation of osteoclasts, thus favoring bone resorption [106]. Vitamin D was also able, in combination with the immunosuppressive drug dexamethasone, to induce tolerogenic DCs, characterized by a defective ability of stimulate T cells, thus suppressing T cell proliferation, IFN-γ, and IL-17 production [107].

In line with these observations, patients with RA show basal serum levels of 25(OH)D lower than healthy controls, and a negative correlation between serum 25(OH)D and RA disease activity was revealed by multiple studies [14, 108, 109]. Notably, vitamin D deficiency also appears as an environmental risk factor for RA [14]. Different studies exploring the association between ultraviolet light exposure and RA risk showed a lower RA risk associated with higher UV-B exposure [110, 111]. In the same vein, vitamin D intake was inversely correlated with the risk of developing RA, at least in postmenopausal women [112]. Furthermore, genetic polymorphisms of VDR (e.g., FokI, TaqI), which may affect vitamin D function, seem to represent additional risk factors in the onset of the disease [19].

All the above-mentioned data suggest a role for vitamin D in the prevention and clinical management of RA. However, available studies on the effect of vitamin D supplementation in RA patients show inconsistent results, revealing, on the one hand, a positive effect of vitamin D on disease risk and activity [113–116] and, on the other hand, no relevant effects on disease outcomes [117]. Studies in animal models and in humans on the effects of vitamin D in RA are summarized in Table 2.

**Table 2:** Studies in animal models and in humans on the effects of vitamin D in RA

Studies in animal models	Studies in humans
↓ development and severity (e.g., hind paw swelling, bone erosion) [103, 104] ↓ autoantibodies production [102] ↓ Th1, Th17, ↑ Treg cells**,** **↓** IL-17**,** IFN-γ [105]	In vitro studies: ↓ TNF-α, IL-1α, IL-1β, IL-6 ↓ RANKL [106] ↑ tolerogenic DCs [107] Clinical studies: ↓ disease risk [114] ↓ disease activity [113, 115, 116] no improvement in RA outcomes [117]

*RANKL* Receptor activator of nuclear factor κβ ligand, *DCs* Dendritic cells. ↓ indicates decrease, ↑ indicates increase

Overall, further studies are needed to clarify the effect of estrogens on vitamin D metabolism and function and to verify whether, as observed in MS, the protective role of estrogens at the systemic level could be mediated, at least partially, by their effect on vitamin D function. Obtained results could allow us to better understand whether therapeutic strategies based on vitamin D could be more effective in females than in males, and whether the decrease of both vitamin D and estrogens, occurred in menopausal women, could favor disease onset and progression.

### Systemic lupus erythematosus

Systemic lupus erythematosus (SLE) is a chronic inflammatory disease with immunological pathogenesis characterized by the involvement of numerous organs including the kidneys and heart, and tissues, such as the skin, muscle, and joints [118, 119]. The symptomatology is represented by the appearance of fever, joint pain, photosensitivity, pleuropericarditis, convulsions, and nephrotic syndrome. The etiology of SLE still remains not fully elucidated but genetic, environmental, and hormonal factors are likely to be involved in disease onset and progression. Profound functional alterations of lymphocytes have been demonstrated including hyperactivity of T and B cells, abnormal T cell-B cell interaction, dysregulated cytokine, and autoantibody production [120–122]. Ninety percent of patients with SLE are females in reproductive age [45]. In addition, women have in general more frequent relapses of disease, whereas men have a late onset of the disease and some more serious comorbidities (e.g., renal comorbidities) [45, 123].

Epidemiological, clinical, and experimental data clearly point to the role of estrogen in the development and clinical manifestations of SLE [21, 124–126]. The change in the female to male ratio with age (i.e., prepuberal age F:M = 2:1, fertile age F:M = 9:1, and postmenopausal age F:M = 3:1) is indicative of the estrogen pathogenetic effect. Accordingly, the use of contraceptive or hormone replacement therapy increases the risk of developing SLE [127]. In addition, differently with that observed in MS and RA, an increase of SLE activity, at least in those women with active disease, was observed during pregnancy due to the Th1 to Th2 shift profile leading to an increase of disease flares and autoantibody production [128, 129]. Finally, as in RA, also in SLE patients, the aromatase activity analyzed in the skin and subcutaneous tissues showed a tendency toward an increase when compared with control subjects [98]. The pathogenetic effect of estrogen in SLE is also supported by in vivo studies in lupus mouse models in which ER deficiency attenuates glomerulonephritis and increases survival [130–132].

As for other autoimmune diseases, also for SLE a role for vitamin D has been suggested in disease development and activity [133–135]. In animal models, a protective effect of vitamin D on disease onset and progression has been observed. In particular, 1,25(OH)2 D3 enhances Treg cells, reduces Th1, Th2, Th17 cells, and autoantibody production, thus ameliorating disease manifestations [136–139]. In humans, vitamin D deficiency is more common in SLE patients than in healthy individuals [140], possibly caused by different reasons such as the reduced sun exposure due to photosensitivity of SLE patients and the occurrence of renal dysfunction, a typical feature of the disease, with consequent inability to convert vitamin D into its active form [133]. In addition, different polymorphisms of the VDR gene have been associated with higher risk for SLE [18].

Low circulating levels of vitamin D have been associated with SLE disease activity as well as extra-musculoskeletal complications such as fatigue, cardiovascular risk, and cognitive impairment [133–135, 141, 142]. A beneficial role of vitamin D in SLE is supported by in vitro studies in peripheral blood mononuclear cells from SLE patients treated with this hormone showing a reduction of anti-DNA antibody production [143] and an inhibition of DC maturation and differentiation [144, 145]. However, the immunomodulatory effects of vitamin D supplementation in SLE patients are still controversial [133–135, 142]. In fact, on the one hand, some studies failed to observe any significant change induced by vitamin D supplementation in SLE disease activity and serology [146–149]. On the other hand, supplementation of vitamin D has been observed to reduce disease activity by an immunosuppressive and anti-inflammatory effect resulting in (i) increase of Treg cells, (ii) decrease of pathogenetic Th1 and Th17 cells, and (iii) decrease of memory B cells and anti-DNA antibodies [113, 150–152]. Studies in animal models and in humans on the effects of vitamin D in SLE are summarized in Table 3.

**Table 3.** Studies in animal models and in humans on the effects of vitamin D in SLE

Studies in animal models	Studies in humans
↓ autoantibodies production [136] ↓ skin ulceration, renal damage [137] ↓ cognitive dysfunction [139] ↓ arthritis [138] ↓ Th1, Th2, Th17 cells, ↑ Treg cells, ↓ IL-4, IL-17, and INF-γ [137]	In vitro studies: ↓ autoantibodies production [143] ↓ maturation and differentiation of DC cells (↓ CD40, MHC-II, and CD86 expression) [145] ↓ IFN-α [144] Clinical studies: ↑ Treg cells, ↓ Th17, Th1 cells, memory B cells [150] ↓ autoantibodies production [113, 150] ↓ disease activity [151] No effects on disease activity and serology [146–149]

*DC* Dendritic cell, *MHC-II* Major histocompatibility complex class II. ↓ indicates decrease, ↑ indicates increase

Interestingly, regarding the cross-talk between vitamin D and estrogen, Kokic and coworkers [153] showed that estrogen, only at low concentration, may have a strong modulating effect on vitamin D function. In fact, studying the potential association between vitamin D and estrogen in SLE, these authors found a significant negative correlation between 25(OH)D and SLE flair in female patients with low serum levels of estrogen. At high values of estrogen, the association between 25(OH)D and SLE flair was not statistically significant, suggesting that vitamin D in this condition lose its protective effect. In this regard, further studies should be carried out to define the molecular mechanism underlying this observation and could open new perspectives in the management of SLE in particular during pregnancy.

## Conclusions

Vitamin D has a key role in modulating immune function with important consequences on health maintenance and disease occurrence, particularly autoimmune disorders. Low serum levels of 25(OH)D have been associated with increased risk of autoimmune disease onset and/or high disease activity. In light of this observation, several studies on the usage of vitamin D for treatment or prevention of these diseases have been carried out. However, optimal doses and duration of supplementation are far to be clearly defined because they may vary between patients, also depending on age and sex. In fact, the elderly population is mostly at risk of developing hypovitaminosis D [154] and gender seems to influences 25(OH)D level by gender-specific determinants such as the time spent outdoors for women and smoking for men [7]. Notably, a cross-talk between vitamin D and estrogen has been suggested by in vivo and in vitro studies, supporting higher protective effects for 1,25(OH)2D in females than in males. However, no information is available regarding fluctuation in estrogen level during women’s life (e.g., pre and postmenopausal period) that should be considered as an important variable in this context. The molecular interaction between vitamin D and estrogen has been well elucidated in MS whereas in RA and in SLE further studies are needed to better characterize this relationship, taking into account the impact of these hormones in disease pathogenesis.

### Perspectives and significance

Overall, this review highlights the importance to expand current knowledge on the immunomodulatory role of 1,25(OH)2D in a sex and gender perspective. This aspect, from a clinical point of view, is noteworthy because an appropriate sex- and age-specific supplementation of vitamin D could represent a useful tool for the treatment of autoimmune diseases. In fact, although the current treatment for these diseases has improved significantly in recent years, particularly with the use of biologics, it is often not disease-modifying and harmful side effects occur [155].

Future clinical studies are needed to define optimal doses of vitamin D for each patient, and how to achieve and maintain these levels taking into account sex and age. In addition, further studies investigating the efficacy of vitamin D in combination with estrogen agonists, at least in those autoimmune diseases in which estrogen display a protective and anti-inflammatory effect such as MS, could lead to new treatment strategies.

## Data Availability

Not applicable.

## Abbreviations

1,25(OH)2D: 1,25-Dihydroxyvitamin D
25(OH)D: 25-Hydroxyvitamin D
ANA: Antinuclear antibody
CNS: Central nervous system
CYP24A1: Cytochrome P450 family 24 subfamily A member 1
CYP27B1: 1α-Hydroxylase
DCs: Dendritic cells
E2: 17-β Estradiol
EAE: Experimental autoimmune encephalomyelitis
IFN: Interferon
IL: Interleukin
MHC-II: Major histocompatibility complex class II
MS: Multiple sclerosis
NK: Natural killer
OVX: Ovarectomized
RA: Rheumatoid arthritis
RANKL: Receptor activator of nuclear factor κβ ligand
SLE: Systemic lupus erythematosus
TGF-β: Transforming growth factor beta
Th: T helper
TLR: Toll-like receptor
TNF-α: Tumor necrosis factor alpha
Treg: T regulatory
VDR: Vitamin D receptor
VDR KO: Vitamin D receptor knockout
